# Phase I trial of pembrolizumab plus vemurafenib and cobimetinib in patients with metastatic melanoma

**DOI:** 10.3389/fonc.2022.1022496

**Published:** 2022-11-23

**Authors:** Saba S. Shaikh, Yan Zang, Janel Hanmer, Hong Wang, Yan Lin, Diwakar Davar, Hassane M. Zarour, John M. Kirkwood, Yana G. Najjar

**Affiliations:** ^1^ Department of Hematology/Oncology, Fox Chase Cancer Center, Philadelphia, PA, United States; ^2^ Department of Medicine, Division of Hematology/Oncology, University of Pittsburgh Medical Center Hillman Cancer Centet, Pittsburgh, PA, United States; ^3^ Department of Medicine, University of Pittsburgh Medical Center, Pittsburgh, PA, United States; ^4^ University of Pittsburgh, School of Public Health, Pittsburgh, PA, United States

**Keywords:** immunotherapy, targeted therapy, BRAF, melanoma, metastatic, clinical trial, immune checkpoint inhibitor, triplet therapy

## Abstract

**Background:**

Preclinical and translational evidence suggest BRAF/MEK inhibitors modulate the tumor microenvironment (TME), providing rationale for combination with immunotherapy.

**Methods:**

This investigator-initiated, phase I trial evaluated pembrolizumab, vemurafenib, and cobimetinib in patients with untreated, BRAFV600E/K mutant advanced melanoma. The first 4 patients received vemurafenib with pembrolizumab, and the next 5 patients received vemurafenib and cobimetinib with pembrolizumab. Primary endpoints: safety and maximum tolerated dose of the triplet.

**Secondary endpoints:**

objective response rate (ORR), progression-free survival (PFS), overall survival (OS), and quality of life (QoL). The trial was closed after enrollment of 9 (planned 30) patients due to dose-limiting toxicity (DLT). Study NCT02818023 was approved by the IRB, and all patients provided informed consent.

**Results:**

Patients received a median of 6 cycles of therapy. 8 of 9 experienced drug-related grade 3/4 AEs. DLTs included dermatitis (n=8), hepatitis (n=1), QTc prolongation (n=1), and arthralgias (n=1 each). QoL assessments identified a clinically significant decrease in self assessed QoL at 1 year compared to baseline (0.38 v 0.43). Median PFS was 20.7 months and median OS was 23.8 months for vemurafenib with pembrolizumab. Median PFS and OS were not reached for patients receiving triple therapy. ORR in the overall cohort was 78% (7/9). 2 patients experienced a complete response, 5 had a partial response, 1 had stable disease, and 1 had progressive disease. 4 patients had ongoing responses at data analysis. Peripheral blood flow cytometry identified significantly decreased PD1 expression on CD4+ T-cells at 3 and 9 weeks compared to baseline, not corresponding to clinical response.

**Conclusions:**

Triple therapy with vemurafenib, cobimetinib and pembrolizumab is associated with high response rates but significant adverse events, leading to early study closure.

## Introduction

The current landscape for management of advanced melanoma includes PD1 inhibitors with or without LAG-3 or CTLA4 inhibitors, and for patients with tumors that harbor BRAFV600E/K mutations, targeted therapy as well. While immunotherapy and targeted therapy each have been shown to improve overall survival in patients with metastatic melanoma (1–3), the recently reported DREAMseq study demonstrated up front combination immunotherapy followed by targeted therapy at the time of progression is associated with a 20% overall survival (OS) benefit compared to up front targeted therapy followed by immunotherapy at progression (4). Preclinical and translational data suggested that BRAF inhibitors (BRAFi) may modulate the tumor microenvironment (TME). After administration of a BRAFi in a BRAF-mutant melanoma model, CD40 ligand and interferon-gamma expression was increased on intratumoral CD4+ tumor-infiltrating lymphocytes (TIL), and regulatory T-cells and myeloid cells were decreased (5), suggesting anti-tumor modulation of the TME. Early data suggested that MEKi may suppress T cell function and RAF/MEKi may inhibit dendritic cell maturation and T cell activation (6, 7). However, further evidence of immune activation was noted when comparing paired patient biopsies at baseline and 10-14 days after treatment with either a BRAFi alone or in combination with a MEK inhibitor (MEKi), which was associated with increased expression of melanoma antigens along with CD8+ TIL (8, 9). Additionally, evidence of immune downregulation has been identified when patients progress on BRAF/MEK inhibition, with a decrease in TIL and antigen expression (10). BRAF inhibition was also associated with an increase in T-cell exhaustion markers TIM-3 and PD1 in tumors of patients with metastatic melanoma (8). Such modification of the TME provided clear rationale for the combination of targeted therapy and immune checkpoint inhibitors.

To date, there have been several reported trials of combinations of checkpoint blockade immunotherapy with targeted therapy. A phase 1 study combining ipilimumab and vemurafenib in patients with metastatic melanoma was closed to accrual due to a high frequency of grade 3 hepatotoxicity (11). Another phase I study evaluated the combination of dabrafenib and ipilimumab, with or without trametinib. The triplet arm of the study closed after 2 of 7 patients experienced colitis complicated by intestinal perforation (12). These early studies highlighted the notable toxicity associated with concurrent administration of immunotherapy and targeted therapy, despite distinct mechanisms of action and individual toxicity profiles that did not otherwise significantly overlap.

In the randomized, phase 2 KEYNOTE-022 trial, patients received either dabrafenib, trametinib and pembrolizumab or dabrafenib, trametinib and placebo, with the primary endpoint of progression-free survival (PFS) (13). Median PFS was higher in the triplet arm (16.0 vs. 10.3 months, HR 0.66 [95% CI 0.4 -1.07], p=0.043), although the trial did not reach the planned benefit for a statistically significant improvement. The investigators speculate this may have been due to differences in the distribution of patients with visceral metastases, of which there were a greater number of patients in the triplet arm. Median duration of response was longer in the triplet arm [18.7 (95% CI 10.1-22.1) vs. 12.5 months (95% CI 6.0-14.1), descriptive HR 0.41]. Grade 3-5 adverse events (AEs) occurred more frequently in the triplet arm (70.0% vs. 45%). The most frequently reported grade 3/4 toxicities were fever (11.7 vs. 5.0%, respectively), increased aspartate aminotransferase (AST; 8.3 vs. 5.0%, respectively), hypertension (8.3 vs. 3.3%, respectively), increased alanine aminotransferase (ALT; 6.7 vs. 5.0%, respectively), increased gamma-glutamyl transferase (GGT; 6.7 vs. 6.7%, respectively), pneumonitis (6.7 vs. 1.7%, respectively), and neutropenia (1.7 vs. 6.7%, respectively). One grade V pneumonitis event was reported in the triplet group.

The randomized, phase 3 COMBI-I trial evaluated the efficacy of spartalizumab, dabrafenib and trametinib compared to placebo, dabrafenib and trametinib as first line treatment of patients with unresectable BRAF mutant melanoma. There was a trend toward improvement in PFS with the triplet combination [16.2 vs. 12 months, HR 0.82 (95% CI, 0.66 to 1.03), one-sided p=0.042], however the study did not meet its primary endpoint. 55% of patients in the triplet arm experienced a treatment-related grade 3 or greater adverse event compared to 33% in the placebo-containing arm. The most frequently reported grade 3/4 events were increased lipase (10 vs. 4%, respectively), increase in blood creatine phosphokinase (7 vs. 5%, respectively), neutropenia (7 vs. 3%, respectively), and fevers (5 vs. 3%, respectively). The most common events that lead to dose modifications included fever, chills, and diarrhea (14).

IMspire150 is the first phase 3 study evaluating a triplet combination that led to regulatory approval for the treatment of BRAF V600E/K mutant metastatic melanoma. Patients with unresectable stage IIIC/IV BRAF V600E/K mutant melanoma were randomized to treatment with atezolizumab, vemurafenib and cobimetinib or placebo, vemurafenib and cobimetinib (15). This regimen was administered in two phases, the first including only vemurafenib/cobimetinib, and the second adding atezolizumab, with reduction in the dosage of vemurafenib/cobimetinib. PFS was prolonged in the atezolizumab-containing arm (15.1 vs. 10.6 months, HR 0.78 [95% CI 0.63-0.97], p=0.025). At the interim OS analysis, 36% of patients had died in the atezolizumab arm compared to 43% in the control arm [HR 0.85 (95% CI 0.64 -1.11), p=0.23]. ORR was similar between the two groups (66.3% vs. 65%) with 15.7% and 17.1% of patients having a CR, respectively. 79% of patients in the triplet arm and 73% of patients in the placebo arm experienced a grade 3/4 AE. The most common grade 3/4 AEs included increased blood creatinine phosphokinase (20 vs. 15%, respectively), increased lipase (20 vs. 21%, respectively), increased ALT (13 vs. 9%, respectively), maculopapular rash (13 vs. 10%, respectively), increased amylase (10 vs. 7%, respectively), and increased AST (8 vs. 4%, respectively). 13% of patients in the triplet arm (vs. 16% in the control arm) stopped all treatment because of AEs.

## Methods

Here, we report the results of a phase 1 study evaluating concurrent pembrolizumab plus vemurafenib and cobimetinib for treatment of advanced BRAF V600E/K mutant melanoma in the first-line setting (NCT02818023). Additional eligibility criteria include, age ≥ 18 years, ECOG 0, 1, or 2, cutaneous or mucosal melanoma, presence of measureable disease, treated and stable brain metastases are permitted, QTc < 480 msThe first four patients received pembrolizumab and vemurafenib (cohort 1), due to early data suggesting that MEKi may be lymphotoxic (16). The protocol was subsequently amended based on emerging data suggesting that MEKi may exert a positive modulatory effect on the TME (17), and the next five patients received pembrolizumab with vemurafenib and cobimetinib (cohort 2). Pembrolizumab was administered at a standard dose of 200 mg every 3 weeks. Patients were enrolled at an initial dose of vemurafenib 720 mg twice daily/cobimetinib 40 mg daily for 21 days in a 28-day cycle. Treatment with pembrolizumab and vemurafenib/cobimetinib began on the same day. The study utilized a modified toxicity probability (mTPI) design. The primary objective was to determine safety and identify the maximum tolerated dose (MTD) of vemurafenib and cobimetinib when administered concurrently with pembrolizumab. MTD was defined as the highest dose with a DLT rate <30%. Patients underwent CT scans at baseline and every 12 weeks to assess treatment response. Secondary endpoints included ORR, PFS, and OS. We planned to accrue 30 patients; however, the trial was closed after enrollment of 9 patients due to an unacceptably high rate of dose-limiting toxicity (DLT). For the mTPI design, the maximum sample size of 30 was determined because it would provide a high probability (>80%) of choosing the correct dose in most likely scenarios. This study was approved by the IRB and all patients provided informed consent.

## Results

**Figure 1:**
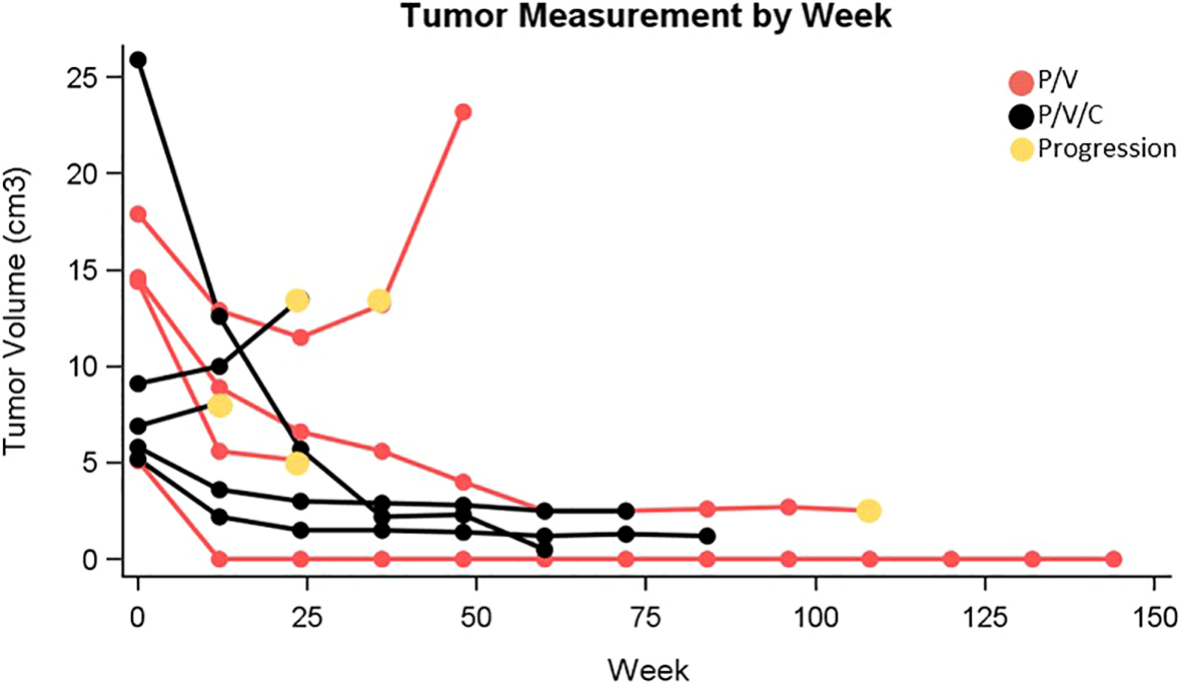
Tumor measurement by week (P/V, pembrolizumab/vemurafenib; P/V/C, pembrolizumab/vemurafenib/cobimetinib).

Patient characteristics can be found in **Table 1**. Patients received a median of 6 cycles of triplet therapy (range: 1-33). In the overall group, 2 patients experienced a complete response, 5 had a partial response, 1 patient had stable disease, and 1 patient had progressive disease as best response. The overall response rate was 78%. One patient in cohort 1 and 3 patients in cohort 2 had ongoing responses at the time of data analysis. Tumor measurements are plotted in **Figure 1**. PFS and OS were estimated and plotted with the Kaplan-Meier method and compared between cohort 1 and cohort 2 using the two-sided log-rank test. Median PFS in the overall group was 30.8 months with 95% CI (2.1, NA). Median PFS was 20.7 months in cohort 1 with 95% CI (6.9, NA), and not reached in cohort 2. Median OS in the overall group was 35.3 months with 95% CI (8.2, NA). Median OS was 23.8 months with 95% CI (8.2, NA) in cohort 1 and not reached in cohort 2. Three patients received subsequent systemic therapy after progression, which included: pembrolizumab, encorafenib/binimetinib, and ipilimumab/nivolumab. One patient enrolled in hospice and did not receive a subsequent line of therapy.

**Table 1:** Patient Characteristics (P/V: pembrolizumab/vemurafenib, P/V/C: pembrolizumab/vemurafenib/cobimetinib).

Patient Characteristic	Overall Group	Cohort 1(P/V)	Cohort 2 (P/V/C)
	( n=9)	(n=4)	(n=5)
Median age(years)	58	65	57
Male sex	6	4	2
Female sex	3	0	3
White race	9	4	5
ECOG			
0	7	2	5
1	2	2	0
Disease stage			
Stage IIIC	1	1	0
Stage IV	8	3	5
Stage, distant metastases			
M1a	2	0	2
M1b	1	1	0
M1c	5	2	3
M1d	0	0	0
Sum of target lesions			
<60 mm	3	1	2
>60 mm	6	3	3
BRAF V600E/K	9	4	5
Median LDH	214	193	214
Prior adjuvant therapy	2	1	1

Eight of nine patients treated with pembrolizumab plus vemurafenib, with or without cobimetinib, experienced DLT. DLTs were defined as any AE that required a dose reduction or discontinuation in the first 3 weeks of treatment. In the vemurafenib and pembrolizumab group, DLTs included hepatitis (n=1), dermatitis (n=3), and arthralgias (n=1). In the vemurafenib with cobimetinib and pembrolizumab group, DLTs included dermatitis (n=5), QTc prolongation (n=1), and arthralgias (n=1). A complete summary of AEs is reported in **Table 2**.

**Table 2:** Adverse events.

Event	Overall (n = 9)		Cohort 1 (n=4)		Cohort 2 (n=5)	
	ALL Grades	Grade ≥ 3	ALL Grades	Grade ≥ 3	ALL Grades	Grade ≥ 3
Any event	8	8	4	3	5	5
Dermatitis	8	7	3	2	5	5
Electrolyte abnormality	7	0	3	2	5	5
Arthralgias	7	2	2	1	5	1
Fatigue	6	0	4	0	2	0
Hepatitis	5	1	2	1	3	0
Fever	4	0	2	0	2	0
Diarrhea	4	2	2	1	2	1
Hypothyroidism	4	0	2	0	2	0
Anemia	4	0	4	0	0	0
Nausea	4	0	4	0	0	0
Burn	3	0	1	0	2	0
Neurotropenia	2	0	1	0	1	0
Thrombocytopenia	2	0	2	0	0	0
Mucositis	2	0	1	0	1	0
Atrial fibrillation	1	0	1	0	0	0
Hypertension	1	0	1	0	0	0
Soft tissue infection	1	0	1	0	0	0
Thrush	1	0	0	0	1	0
Pneumonitis	1	0	1	0	0	0
Adrenal Insufficiency	1	1	0	0	1	1
Edema	1	0	0	0	1	0

Quality of life assessments were collected at baseline, 9 weeks, 6 months, and 1 year. These assessments evaluated patient-reported anxiety, depression, cognitive function, fatigue, pain, physical function, sleep, and social roles using the PROMIS-29 Profile v2.0 and the Cognitive Function short form 4a (18, 19). These PROMIS measures have a mean of 50 with a standard deviation of 10 in the US general population. These assessments identified worsening depression (53.6 vs. 50.6), decreased cognitive function (50.2 vs. 54.4), and increasing fatigue (51.2 vs. 50.3) at 1 year compared to baseline. Anxiety, pain, physical function, sleep, and social roles were not significantly different. A PROPr score of health utility was also calculated in which a value of 1 corresponds to full health and a score of 0 corresponds to dead (20). The assessments identified a clinically significant decrease in average health utility at 1 year compared to baseline (0.38 vs. 0.43). Of note, one patient had evidence of PD at the first scan, and no further assessments were collected for that patient.

Blood samples were collected at baseline, 3 weeks, and 9 weeks, and tumor biopsy samples were collected at baseline and at week 3, when feasible. Peripheral blood flow cytometry was performed to assess CD4, CD8, FOXP3, Ki67, PD-1, and LAG3. PD1 expression on the CD4+ T-cells was significantly decreased at 3 weeks compared to baseline (0.9 vs 2.8, p=0.0339 with paired t-test) and remained decreased at 9 weeks (1.1 vs 2.8, p=0.0282) without a significant increase from week 3 (p=0.5574), which may suggest decreased T-cell exhaustion. This did not correspond to clinical response data. The remainder of the flow data did not identify statistically significant differences across the assessed time points. PD-L1 testing was also performed on 6 paired tumor samples, and no significant association was identified between PD-L1 expression at baseline and clinical outcomes.

## Discussion

Despite the preclinical and translational evidence for tumor immune modification with BRAF/MEK inhibitors and a PFS of 15.1-16.2 months in the 3 largest reported triplet-therapy trials, the toxicity incurred with triplet therapies has been challenging from a practical standpoint (13–15). Triplet therapies were associated with significant increases in grade 3/4 adverse events compared to doublet therapies in both KEYNOTE-022 (70% vs. 45%) and COMBI-I (55% vs. 33%). There was also a slight increase in grade 3/4 AEs in the triplet arm in IMspire150 (79% vs. 73%). Of note, the control arm in IMspire150 had a higher AE rate compared to the cohort of patients receiving vemurafenib and cobimetinib in coBRIM, in which 60% of patients experienced a grade 3/4 AE (2). Our study adds additional toxicity data for triplet therapy, with 8 of 9 patients experiencing a DLT. Of note, the maximum dose of vemurafenib and cobimetinib administered in this trial was 720 mg twice daily/40 mg daily, which is comparable to the reduced dose of BRAFi in the triplet arm in IMspire150 from cycle 2 onwards. In cycle 1 of IMspire150, pts in the triplet arm received a 3 week-lead in with vemurafenib 960 mg twice-daily and cobimetinib at 60 mg daily, which may have contributed to the difference in adverse event profile compared to our study. The difference may also be related to the differences in immune checkpoint inhibitor. This study utilized pembrolizumab, a PD-1 inhibitor, compared to atezolizumab, a PD-L1 inhibitor. A meta-analysis of 125 clinical trials identified higher rate of grade 3 AEs with PD-1 inhibitors compared to PD-L1 inhibitors, raising the question of interchangeability of these agents with respect to toxicity (21). In addition to significant toxicity, patients reported a significant decrease in overall health utility at 1 year compared to baseline, which may be driven by depression, cognitive function, and fatigue. Here, we report median PFS of 30.8 months in the overall group, which is increased compared to the PFS reported in IMspire 150, which was 15.1 months in the triplet group compared to 10.6 months in control. Of note, the control arm of vemurafenib and cobimetinib had a lower PFS compared to the coBRIM trial, which reported a median PFS of 12.3 months in the doublet arm (vs. 7.2 months, HR 0.58 [95% CI 0.46-0.72], p<0,0001). The PFS benefit seen in IMspire 150 may be related to the increased duration of response in the triplet arm (21.0 vs. 16.0 months) given that the ORR was similar (66% vs. 65%). Notably, IMspire150 compared the triplet regimen to BRAF/MEK inhibition, and how the efficacy data compares to anti-PD1 therapy with or without anti-CTLA4 or anti-LAG3 therapy is not known. Furthermore, encorafenib and binimetinib have since been approved in the metastatic setting, with higher PFS and overall improved tolerability than reported with other targeted therapies (22). This raises the question of whether combination therapy of anti-PD1 and newer BRAF/MEK inhibitors may be better tolerated, and studies of this question are ongoing (NCT04657991, NCT04511013). Our study of vemurafenib and cobimetinib with pembrolizumab had a high ORR but closed early due to high rates of grade 3/4 AEs. In addition, the survival benefit of up-front combination immunotherapy compared to targeted therapy further puts into question which patients would benefit from triplet combination therapy (23). Overall, given the significant toxicities incurred with triplet therapy and modest PFS improvements, physicians must think carefully about which patients are best served with this treatment strategy.

## Data availability statement

The raw data supporting the conclusions of this article will be made available by the authors, without undue reservation.

## Ethics statement

The studies involving human participants were reviewed and approved by University of Pittsburgh Medical Center IRB. The patients/participants provided their written informed consent to participate in this study.

## Author contributions

SS: Data acquisition, analysis and interpretation, manuscript writing. YZ: Data acquisition, analysis and interpretation. JH: Analysis and interpretation, critical manuscript revisions. HW, YL, DD, HZ, JK: Analysis and interpretation, critical manuscript revisions. YN: Conception and study design, analysis and interpretation, manuscript writing. All authors contributed to the article and approved the submitted version.

## Funding

This project was funded by Merck. In addition, this project is funded, in part, under a Grant with the Pennsylvania Department of Health. The Department specifically disclaims responsibility for any analyses, interpretations or conclusions.

## Conflict of interest

Author DD reports the following disclosures: Arcus, Checkmate Pharmaceuticals, CellSight Technologies, Immunocore, Merck, Tesaro/GSK research support; Checkmate Pharmaceuticals, Finch, Shionogi, Vedanta Biosciences consulting; Vedanta Biosciences scientific advisory board. CE Speakers’ Bureau: Medical Learning Group MLG, Clinical Care Options CCO. Intellectual Property: US Patent 63/124,231, “Compositions and Methods for Treating Cancer”, Dec 11, 2020; US Patent 63/208,719, “Compositions and Methods For Determining Responsiveness to Immune Checkpoint Inhibitors ICI, Increasing Effectiveness of ICI and Treating Cancer”, June 9, 2021. Author HZ reports the following disclosures: Bristol-Myers Squibb, Checkmate Pharmaceuticals, and GlaxoSmithKline research support and Bristol-Myers Squibb, Checkmate Pharmaceuticals, GlaxoSmithKline, and Vedanta consulting. Author JK reports the following disclosures: Amgen, Bristol-Myers Squibb, Castle Biosciences, Checkmate Pharmaceuticals, Immunocore LLC, Iovance, and Novartis research support and Amgen, BristolMyers Squibb, Checkmate Pharmaceuticals, and Novartis consulting. Author YN reports the following disclosures: Merck, Pfizer, and Bristol-Myers Squibb research support. Array Biopharma, Merck, Novartis, InterVenn Bio consulting/scientific advisory board. Pfizer, Immunocore speaker’s bureau. CE Speakers’ Bureau: Medical Learning Group MLG.

The remaining authors declare that the research was conducted in the absence of any commercial or financial relationships that could be construed as a potential conflict of interest.

## Publisher’s note

All claims expressed in this article are solely those of the authors and do not necessarily represent those of their affiliated organizations, or those of the publisher, the editors and the reviewers. Any product that may be evaluated in this article, or claim that may be made by its manufacturer, is not guaranteed or endorsed by the publisher.
